# Allosteric Control of Substrate Specificity of the *Escherichia coli* ADP-Glucose Pyrophosphorylase

**DOI:** 10.3389/fchem.2017.00041

**Published:** 2017-06-19

**Authors:** Ana C. Ebrecht, Ligin Solamen, Benjamin L. Hill, Alberto A. Iglesias, Kenneth W. Olsen, Miguel A. Ballicora

**Affiliations:** ^1^Department of Chemistry and Biochemistry, Loyola University ChicagoChicago, IL, United States; ^2^Laboratorio de Enzimología Molecular, Instituto de Agrobiotecnología del Litoral (UNL-CONICET), CCT CONICETSanta Fe, Argentina

**Keywords:** allosteric regulation, effectors interaction, glycogen biosynthesis, substrate promiscuity

## Abstract

The substrate specificity of enzymes is crucial to control the fate of metabolites to different pathways. However, there is growing evidence that many enzymes can catalyze alternative reactions. This promiscuous behavior has important implications in protein evolution and the acquisition of new functions. The question is how the undesirable outcomes of *in vivo* promiscuity can be prevented. ADP-glucose pyrophosphorylase from *Escherichia coli* is an example of an enzyme that needs to select the correct substrate from a broad spectrum of alternatives. This selection will guide the flow of carbohydrate metabolism toward the synthesis of reserve polysaccharides. Here, we show that the allosteric activator fructose-1,6-bisphosphate plays a role in such selection by increasing the catalytic efficiency of the enzyme toward the use of ATP rather than other nucleotides. In the presence of fructose-1,6-bisphosphate, the *k*_cat_/*S*_0.5_ for ATP was near ~600-fold higher that other nucleotides, whereas in the absence of activator was only ~3-fold higher. We propose that the allosteric regulation of certain enzymes is an evolutionary mechanism of adaptation for the selection of specific substrates.

## Introduction

Enzymes exhibit a remarkable capacity to evolve and acquire new functions for the adaptation of the organism to its environment. A clear example is the fast evolution of bacteria to drug resistance strains (Khersonsky and Tawfik, 2010). The connection between promiscuity and protein evolution was first formalized in 1976 by Jensen, who proposed that ancient enzymes possessed a broad substrate specificity (Jensen, 1976). These few rudimentary enzymes acted on multiple substrates to afford a wider range of metabolic capabilities (Jensen, 1976; Khersonsky and Tawfik, 2010). Later, enzyme families grew by gene duplication coupled with the acquisition of advantageous new functions (Nam et al., 2012; Pandya et al., 2014; Donertas et al., 2016). Thus, the identification of promiscuity (also called ambiguity when the chemistry is the same, Jensen, 1976; Pandya et al., 2014) provides important information about the evolutionary and mechanistic relationships among members of the same enzyme superfamily. Several studies have focused on the structural and mechanistic aspects of promiscuity, its role in the evolution of new functions, and its practical implications (Khersonsky and Tawfik, 2010; Pandya et al., 2014). However, it is important to understand how the promiscuity and its physiological consequences are avoided *in vivo*. This is physiologically relevant because the enzyme specificity for substrates controls metabolic flow by sorting metabolites into distinct paths. In this work we studied how the promiscuity of the ADP-glucose pyrophosphorylase (glucose-1-phosphate adenylyltransferase, EC 2.7.7.27; ADP-Glc PPase) is controlled.

ADP-Glc PPase and other sugar nucleotide pyrophosphorylases belong to the nucleotidylyltransferase family. Despite large diversity existing at primary and quaternary structure levels, nucleotidylyltransferases share some common features: the catalytic domain has the same GT-A fold (Singh et al., 2012), they require a divalent cation for catalytic activity (generally Mg^2+^), and they share conserved residues and motifs in the active site (Fuhring et al., 2013). Analysis of known structures showed that conserved residues are in homologous locations in the active site binding the sugar nucleotide (Jin et al., 2005). For example, the glycine rich GXGXRL loop is present in most of the sugar nucleotide pyrophosphorylases described so far, and it was identified as the site of nucleoside triphosphate (NTP) binding (Brown et al., 1999; Sivaraman et al., 2002; Jin et al., 2005; Koropatkin et al., 2005; Maruyama et al., 2007; Steiner et al., 2007; Pelissier et al., 2010). ADP-Glc PPase has a distinct characteristic of being allosterically regulated by small effector molecules (Ballicora et al., 2003, 2004). The enzyme synthesizes ADP-Glc, from ATP and glucose-1-phosphate (Glc-1P), which is used for the production of storage polysaccharides (glycogen and starch) in bacteria and plants.

Previous works reported that ADP-Glc PPase has a certain degree of promiscuity toward the substrates (Preiss et al., 1966; Lapp and Elbein, 1972; Hill et al., 1991; Machtey et al., 2012; Cereijo et al., 2016), but the control of this promiscuity has not been studied. Here, we analyzed the use of alternative substrates by the ADP-Glc PPase from *Escherichia coli*, and how the activators, fructose-1,6-bisphosphate (Fru-1,6-bisP) and pyruvate (Pyr), play a key role in substrate selection. We observed that Fru-1,6-bisP markedly increased the catalytic efficiency of the enzyme toward the use of ATP and Glc-1P, restricting its promiscuous behavior. Computational studies suggest why alternative nucleotides have relatively low *K*_m_ and *k*_cat_ values.

## Materials and methods

### Chemicals

Glc-1P, Gal-1P, GlcN-1P, ATP, ITP, UTP, CTP, GTP, ADP-Glc, UDP-Glc, GDP-Glc, inorganic pyrophosphate (PPi) and Fru-1,6-bisP were obtained from Sigma-Aldrich (St. Louis, MO, USA). All other reagents were of the highest quality available.

### Expression and purification of recombinant proteins

Recombinant *E. coli* ADP-Glc PPase and its mutant W113A were obtained from *E. coli* BL21 (DE3) cells (Novagen, Madison, WI, USA) transformed with pETEC and pETEC W113A vectors as previously described (Figueroa et al., 2011). Expression of the pETEC plasmid (and pETEC W113A), as well as purification of the recombinant enzymes were performed as previously described (Ballicora et al., 2002; Figueroa et al., 2011).

### Protein assay and gel electrophoresis

Protein concentration of the purified enzymes was estimated by the UV absorbance at 280 nm using an extinction coefficient of 1.0 ml mg^−1^ cm^−1^(Figueroa et al., 2011). Protein electrophoresis under denaturing conditions (SDS-PAGE) was performed as described previously (Laemmli, 1970). Following electrophoresis, protein bands were visualized by staining with Coomassie Brilliant Blue R-250.

### Enzyme activity assays

Activities were determined at 37°C in both directions: NDP-Glc synthesis (assay A) and pyrophosphorolysis (assay B). In all of the assay procedures, one unit of activity (U) is defined as the amount of enzyme catalyzing the formation of 1 μmol of product per min, under conditions described above in each case. Unless otherwise specified, conditions for the different assays were as follows.

*Assay A*. Synthesis of ADP-Glc (UDP-Glc, CDP-Glc and GDP-Glc) was assayed by following the formation of Pi (after hydrolysis of PPi by inorganic pyrophosphatase) by the colorimetric method previously described (Fusari et al., 2006). Reaction mixtures contained 50 mM MOPS-HCl pH 8.0, 10 mM MgCl_2_, 1.5 mM ATP (ITP, UTP, CTP, or GTP), 0.2 mg/ml BSA, 0.0005 unit/μl yeast inorganic pyrophosphatase, the appropriate enzyme dilution and 1 mM Fru-1,6-bisP (when necessary). Assays were initiated by the addition of 1.5 mM Glc-1P in a total volume of 50 μl. Reaction mixtures were incubated for 10 min at 37°C and terminated by adding reactive malachite green (Fusari et al., 2006). The complex formed with the released Pi was measured at 630 nm with an ELISA (enzyme-linked immunosorbent assay) EMax detector (Molecular Devices). The same procedure was followed for the analysis of enzyme promiscuity toward sugar-1P, except that in this case the different monosaccharide-1Ps were included in the assay mixture and reactions were started with 1.5 mM ATP.

For alternative cofactors for ADP-Glc PPase, assay were studied by performing the reaction with different metals in absence of inorganic pyrophosphatase, during 10 min at 37°C; reaction was stopped with boiling water and 10 mM EDTA. Then, Mg^2+^ and inorganic pyrophosphatase were added to the samples, the reaction mixture was further incubated for 10 min at 37°C, and the reaction was stopped with malachite green as described above. This was necessary due to the essential requirement of Mg^2+^ for activity of inorganic pyrophosphatase.

*Assay B*. For activity in pyrophosphorolysis direction, the enzyme was assayed with a coupled-enzyme spectrophotometric assay. The reaction mixture contained 80 mM HEPPS-NaOH pH 8.0, 10 mM Mg^2+^, 1 mM Fru-1,6-bisP (when necessary), 1 mM ADP-Glc (or 10 mM UDP-Glc or 10 mM GDP-Glc), 0.6 mM NAD^+^, 0.01 mM Glc-1,6-bisP, 2 U/ml Phosphoglucomutase, 2 U/ml Glc-6P dehydrogenase, 0.2 mg/ml BSA and enzyme in a total volume of 80 μl. The reaction was initiated with 1 mM PPi and absorbance at 340 nm was followed for 10 min every 15 s at 37°C using a BioTek EL808 microplate reader (Winooski, VT, USA). For inhibition assays with the different nucleotides, activity was measured in at sub-saturating concentration of ADP-Glc (0.2 mM) and increasing concentration of NTP-Mg^2+^.

### Kinetic studies

Kinetic assays were performed using specified concentrations and conditions for all reaction mixture components. Saturation curves were performed by assaying the respective enzyme activities at saturating levels of a fixed substrate and different concentrations of the variable substrate (or effector). The experimental data was plotted as enzyme activity (U/mg) versus substrate (or effector) concentration (mM), and the kinetic constants were determined by fitting the data to the Hill equation as described elsewhere (Ballicora et al., 2007). Fitting was performed with the Levenberg-Marquardt nonlinear least-squares algorithm provided by the computer program Origin™ 8.0. Hill plots were used to calculate the Hill coefficient (*n*), the maximal velocity (*V*_max_), and the kinetic constants that correspond to the activator, substrate, or inhibitor concentrations giving 50% of the maximal activation (*A*_0.5_), velocity (*S*_0.5_), or inhibition (*I*_0.5_). Values of activity in s^−1^were calculated considering the molecular mass of the enzyme monomer (50 kDa). All kinetic constants are the means of at least three independent sets of data, which were reproducible within ± 10%.

### Promiscuity index

Promiscuity index (*I*) was calculated according to (Nath and Atkins, 2008):

$$I=-\frac{1}{\log N}\sum_{i=1}^{N}\frac{e_{i}}{\sum_{j=1}^{N}e_{j}}\log\frac{e_{i}}{\sum_{j=1}^{N}e_{j}}$$

Where *N* is the total of all the substrates and *e* = *k*_cat_/*S*_0.5_. Values of *e* used are listed in Tables S1–S3.

### Computational methods

The *E. coli* ADP-Glc PPase structure with NTP substrates was constructed using the following templates: the crystal structure of the small subunit of the *Solanum tuberosum* in complex with ATP (PDB ID: 1YP3), and *Agrobacterium tumefaciens* ADP-Glc PPase (PDB ID: 3BRK) (Jin et al., 2005; Cupp-Vickery et al., 2008). An *E. coli* homotetramer model was generated using Modeler (Sali and Blundell, 1993; Eswar et al., 2007). The structure with the NTP substrate had two ATP molecules in two of the four subunits, subunit A and C. ATP, UTP, GTP, CTP, and ITP substrates were constructed and placed in the predicted active site of subunits A and C. Two Mg^2+^ ions were placed in the active site of all four subunits. The protein, substrates and ions were placed in a TIP3 water box that extended at least 10 Å beyond the protein in all directions and 0.1 M NaCl adjusted to neutralize the charge in the water box. Placement of the substrates, ions, and the generation of the water box were all assembled using the molecular graphics program VMD (Humphrey et al., 1996). The molecule was then brought to equilibrium using the molecular dynamics program NAMD (Phillips et al., 2005). The equilibration procedure involved energy minimization with and without restraints on the protein coordinates (3,000 steps each), slow heating from 10 to 310 K (30,000 steps), pressure and temperature equilibration using a Langevin piston (10,000 steps) and unrestrained dynamics for 100,000 steps before data was acquired. Periodic boundary conditions were used. The cutoffs for non-bonding (van der Waals and electrostatic) interactions were 12 Å. The switch distance was 10 Å, and 1.0 1-4 scaling factor was used. All calculations were done using CHARMM 27 parameters (Mackerell, 2004). Molecular dynamic simulations of 10 ns were created using NAMD for each ADP-Glc PPase and NTP substrate (Phillips et al., 2005).

A second model of the *E. coli* ADP-Glc PPase was generated using the crystal structure of the small subunit of the potato tuber ADP-Glc PPase complex with ADP-Glc (PDB ID: 1YP4) and again the *A. tumefaciens* ADP-Glc PPase (PDB ID: 3BRK) as templates (Jin et al., 2005; Cupp-Vickery et al., 2008). Only subunit B of the homotetramer contained the ADP-Glc substrate. The ADP-glucose structure was used as a template to form the other NDP-Glc structures. Two Mg^2+^ ions were again placed in each of the subunits; the protein, NDP-Glc substrate, and ions were placed in a water box, brought to equilibrium, and 10 ns of molecular dynamics were collected following the procedure described above.

Using the Volmap tool from VMD, volumetric maps based on the weighted atomic density of each point were generated with a resolution of 0.1 Å. Average structures of each NTP and NDP-glucose were calculated using the last 5 ns of each molecular dynamic simulation to ensure the structures were fully equilibrated. In that part of the simulation, the simulated structures already stabilized to an RMSD of ~1.8 to ~2.2 Å from the original one (Figure S1).

## Results

**Figure 1:**
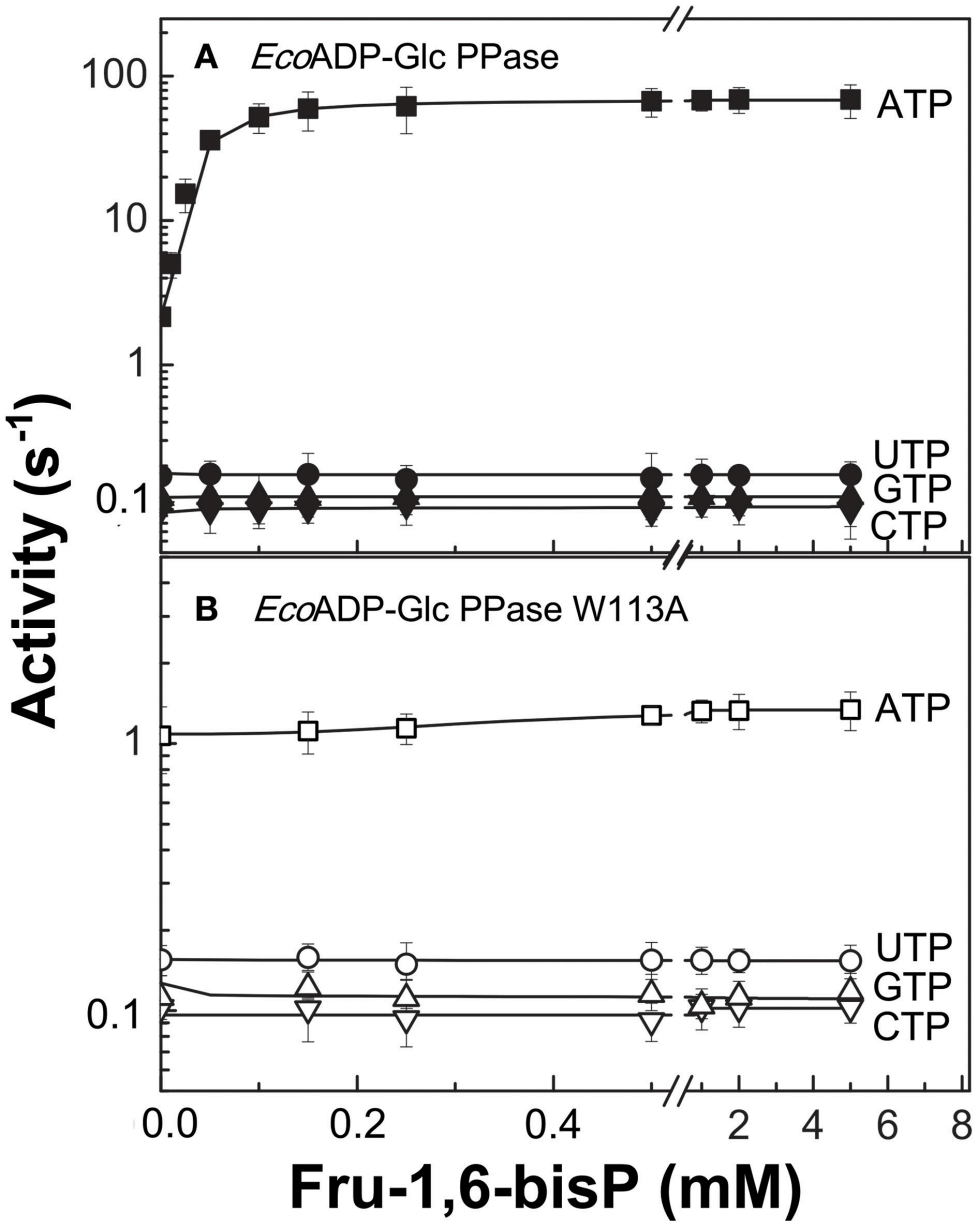
Nucleotide dependent effect of Fru-1,6-bisP on ADP-Glc PPase activation. Activity of **(A)** *E. coli* ADP-Glc PPase wild type and **(B)** W113A mutant with alternative nucleotide (2 mM) as substrates. Error bars are SD of three independent sets of data.

We characterized the *E. coli* ADP-Glc PPase substrate specificity for ATP (forward direction) and for ADP-Glc (reverse direction). The enzyme was able to use other NTPs (Table 1) and NDP-Glc (Table 2) less efficiently. As described before (Ballicora et al., 2003, 2007; Figueroa et al., 2011), Fru-1,6-bisP increased *k*_cat_ and reduced the *S*_0.5_values for the substrates. Conversely, when the enzyme used an alternative NTP (UTP, CTP, and GTP) the allosteric activator had no effect on the *k*_cat_ (Figure 1A, Table 1) or the *S*_0.5_ of any of these alternative substrates (Table 1). In the absence of Fru-1,6-bisP the *k*_cat_ measured with UTP, GTP or CTP was ~70-fold lower than that observed with ATP, whereas in the presence of the effector the maximum activity was at least 400-fold higher with ATP than with the other NTPs (Table 1). But the greater effect of Fru-1,6-bisP was to increase the apparent affinity for ATP. Therefore, the best criterion of comparison is the *k*_cat_/*S*_0.5_ ratio for the native and alternative substrates (analogous to the catalytic efficiency or *k*_cat_/*K*_m_ for hyperbolic kinetics). In the absence of Fru-1,6-bisP, the *k*_cat_/*S*_0.5_ for all four more common NTPs were in the same range, but in its presence the ratio for ATP dramatically increased (~200-fold) (Figure S2A and Table 1). A particularly interesting analog of ATP is ITP because the only structural difference is a keto group instead of a primary amino group. The results with ITP were similar to the ones found for the other alternative NTPs (Table 3).

**Table 1:** Kinetic parameters for substrates of the *E. coli* ADP-GlcPPase in the synthesis direction.

		**CONTROL**				+ **Fru-1,6-bisP**			
**Synthesis of**	**Substrate**	***S*_0.5_ (mM)**	***n***	***k*_cat_ (s^−1^)**	***k*_cat_/*S*_0.5_(s^−1^ mM^−1^)**	***S*_0.5_ (mM)**	***n***	***k*_cat_ (s^−1^)**	***k*_cat_/*S*_0.5_(s^−1^ mM^−1^)**
ADP-Glc	ATP	11 ± 4	1.3 ± 0.1	10.2 ± 0.3^*^	1	0.32 ± 0.02	2.3 ± 0.2	56 ± 1	177
	Glc-1P	0.54 ± 0.04	1.2 ± 0.2		18	0.03 ± 0.01	1.2 ± 0.1		1888
	Mg^2+^	4.0 ± 0.1	1.7 ± 0.2		2.5	1.9 ± 0.2	2.8 ± 0.3		29
UDP-Glc	UTP	0.40 ± 0.06	1.4 ± 0.2	0.14 ± 0.01	0.3	0.40 ± 0.04	1.4 ± 0.2	0.14 ± 0.02	0.3
	Glc-1P	0.38 ± 0.03	1.3 ± 0.2		0.3	0.36 ± 0.02	1.1 ± 0.1		0.4
	Mg^2+^	3.2 ± 0.3	2.3 ± 0.2		0.04	3.8 ± 0.4	2.2 ± 0.3		0.03
GDP-Glc	GTP	0.51 ± 0.03	1.5 ± 0.1	0.10 ± 0.01	0.2	0.35 ± 0.02	1.6 ± 0.2	0.10 ± 0.01	0.2
	Glc-1P	0.22 ± 0.03	1.4 ± 0.2		0.4	0.16 ± 0.01	1.1 ± 0.1		0.6
	Mg^2+^	2.8 ± 0.2	1.9 ± 0.2		0.03	2.5 ± 0.1	2.3 ± 0.2		0.03
CTP-Glc	CTP	0.29 ± 0.02	1.4 ± 0.2	0.08 ± 0.01	0.2	0.25 ± 0.01	1.3 ± 0.2	0.08 ± 0.01	0.3
	Glc-1P	0.37 ± 0.03	1.2 ± 0.2		0.2	0.34 ± 0.03	1.1 ± 0.1		0.2
	Mg^2+^	2.9±.03	2.0 ± 0.1		0.02	2.7 ± 0.1	2.1 ± 0.2		0.03

* *k_cat_ calculated from saturation plot of ATP*.

**Table 2:** Kinetic parameters for substrates of the *E. coli* ADP-GlcPPase in the pyrophosphorolysis direction.

		**CONTROL**				+ **Fru-1,6-bisP**			
**Pyrophosphorolysis of**	**Substrate**	***S*_0.5_ (mM)**	***n***	***k*_cat_ (s^−1^)**	***k*_cat_/*S*_0.5_(s^−1^ mM^−1^)**	***S*_0.5_ (mM)**	***n***	***k*_cat_ (s^−1^)**	***k*_cat_/*S*_0.5_(s^−1^ mM^−1^)**
ADP-Glc	ADP-Glc	0.42 ± 0.04	1.3 ± 0.1	12 ± 2	27	0.20 ± 0.02	2.1 ± 0.2	110 ± 6	550
	PPi	0.50 ± 0.04	2.7 ± 0.3		23	0.07 ± 0.01	1.0 ± 0.1		1572
	Mg^2+^	5.4 ± 0.5	1.8 ± 0.2		2	2.2 ± 0.2	2.0 ± 0.2		50
UDP-Glc	UDP-Glc	2.4 ± 0.3	1.0 ± 0.1	0.41 ± 0.05	0.1	2.2 ± 0.2	0.9 ± 0.1	0.40 ± 0.03	0.1
	PPi	0.54 ± 0.04	1.8 ± 0.1		0.8	0.50 ± 0.05	1.8 ± 0.1		0.8
	Mg^2+^	5.5 ± 0.5	1.7 ± 0.1		0.1	5.2 ± 0.2	2.1 ± 0.2		0.1
GDP-Glc	GDP-Glc	4.9 ± 0.4	2.3 ± 0.3	>0.33	0.1	4.9 ± 0.3	1.7 ± 0.2	>0.30	0.1
	PPi	>1	N/A		N/A	>1	N/A		N/A
	Mg^2+^	4.4 ± 0.5	1.9 ± 0.2		0.1	4.7 ± 0.3	2.6 ± 0.4		0.1

**Table 3:** Kinetic parameters for ITP.

	**CONTROL**	**+ Fru-1,6-bisP**
*S*_0.5_(mM)	0.55 ± 0.04	0.50 ± 0.03
*n*	2.6 ± 0.4	1.8 ± 0.2
*k*_cat_ (s^−1^)	0.55 ± 0.01	0.90 ± 0.01
*k*_cat_/*S*_0.5_ (s^−1^ mM^−1^)	1	1.8
(*k*_cat_/*S*_0.5_)_ATP_/(*k*_cat_/*S*_0.5_)^*^_ITP_	1	99

* *Data for ATP come from Table 1. Concentrations of Glc-1P (1.5 mM) and Mg^2+^ (10 mM) were saturating and assays were done as described in Materials and Methods*.

To further explore the functional relevance of Fru-1,6-bisP on the use of alternative NTPs, we tested the previously characterized mutant W113A (Figueroa et al., 2011). In *E. coli* ADP-Glc PPase, the W113 was postulated to trigger the enzyme activation upon Fru-1,6-bisP binding. Mutation of this residue disrupted the communication between the regulatory site and the catalytic site yielding an enzyme insensitive to the activator (Figueroa et al., 2011, 2013; Hill et al., 2015). W113A, even in the presence of the activator, had a promiscuous behavior similar to the WT in the absence of the activator (Figure 1). The fact that this mutant is insensitive to Fru-1,6-bisP confirms that without communication between the regulatory site and the catalytic site the enzyme loses substrate selectivity (Figure 1B). All of these results indicate that Fru-1,6-bisP increases specificity for ATP.

**Figure 2:**
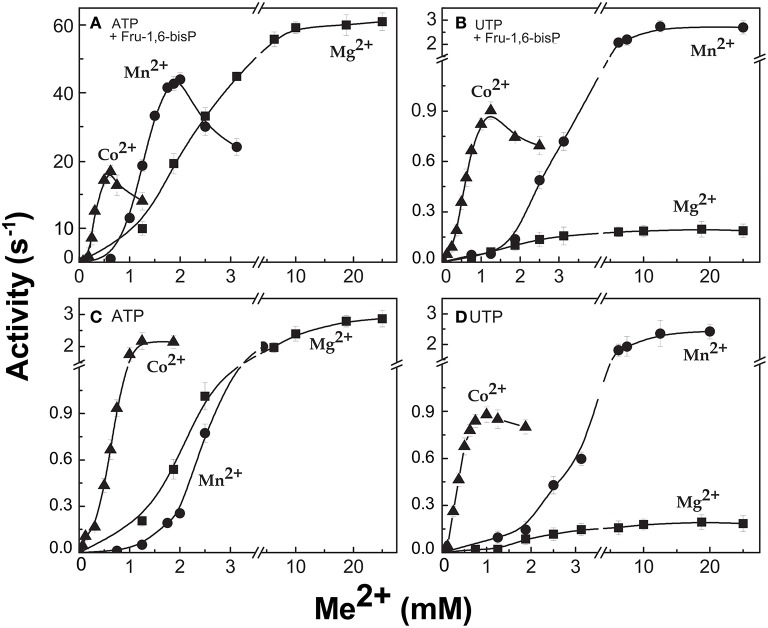
ADP-Glc PPase activity with different divalent cations as cofactor of the reaction. Activity was measured in presence **(A,B)** and absence **(C,D)** of Fru-1,6-bisP. Error bars are SD of three independent sets of data.

When the enzyme used ATP as a substrate, Fru-1,6-bisP also increased the *k*_cat_/*S*_0.5_for the second substrate Glc-1P and Mg^2+^ cofactor (~100-fold and ~10-fold, respectively, Table 1). But, Fru-1,6-bisP did not have this effect when other NTPs were used. These results indicate that the activator not only selects the nucleotide substrate but also increases the *k*_cat_/*S*_0.5_ of the sugar phosphate. This could be an indirect effect because the sugar phosphate binds second according with the sequential Iso Ordered Bi Bi kinetic mechanism previously determined for the enzyme (Kleczkowski et al., 1993). To test the “selective” effect of Fru-1,6-bisP on the second substrate and the cofactor, we decided to assay alternative sugar-1Ps and divalent cations. The three metals Co^2+^, Mn^2+^, and Mg^2+^ were effective cofactors in reactions with both ATP and UTP. In assays performed with ATP, Fru-1,6-bisP improved the *k*_cat_/*S*_0.5_ for the metals by two orders of magnitude (Figure S2C). The effect of the activator was on *k*_cat_, especially for Mg^2+^ (Figures 2A,C). On the other hand, the enzyme using UTP reached a higher *k*_cat_ with Mn^2+^ and Co^2+^ than with Mg^2+^, but the enzyme was insensitive to Fru-1,6-bisP (Figures 2B,D). It has been described that ADP-Glc PPase is able to use different sugar-1Ps, but with much less efficiency (Hill et al., 1991). To study the effect of Fru-1,6-bisP on the selectivity for this substrate, we assayed the enzyme with galactose-1P (Gal-1P) and glucosamine-1P (GlcN-1P). The allosteric activator increased the *k*_cat_/*S*_0.5_ of all sugar-phosphates (Figure S2B). Using Glc-1P as the substrate achieved the greatest *k*_cat_/*S*_0.5_, but in relative terms, the highest increase was observed with GlcN-1P.

**Figure 3:**
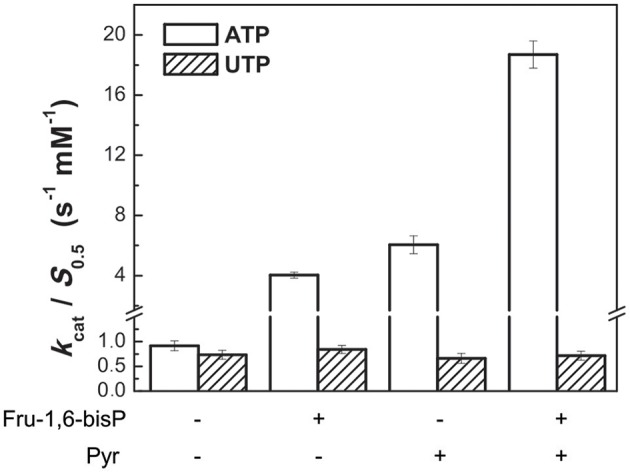
Activators effect on ADP-Glc PPase catalytic efficiency. Activity was measured when the enzyme used alternative ATP (white bars) or UTP (oblique line bars) as substrate in presence of allosteric effectors. Error bars are SD of three independent sets of data.

It was reported that Pyr modulates the activity of *E. coli* ADP-Glc PPase synergistically with Fru-1,6-bisP (Asencion Diez et al., 2014). We assayed, in presence of different concentrations of these synergistic activators, the enzyme with UTP and ATP. Non-saturating concentrations of Fru-1,6-bisP (10 μM) increased the *k*_cat_/*S*_0.5_ for ATP by 4.5-fold, but it had no effect on UTP (Figure 3). In the presence of 20 mM Pyr, the *k*_cat_/*S*_0.5_ was increased only when the enzyme used ATP as the substrate (~7-fold higher). In addition, there was a synergic effect on selectivity: in the presence of Pyr and non-saturating concentrations of Fru-1,6-bisP the *k*_cat_/*S*_0.5_ was improved ~20-fold for ATP. In reactions with UTP no improvement was observed.

Similar behavior was observed in the pyrophosphorolysis direction. The enzyme activity for the pyrophosphorolysis of ADP-Glc was increased by the activator Fru-1,6-bisP, but with UDP-Glc and GDP-Glc the *k*_cat_ remained the same (Table 2). Since ADP-Glc PPase was able to use different NTPs as substrates, we hypothesized that these could inhibit the pyrophosphorolysis reaction catalyzed by the enzyme. This would be an indirect way to assess the nucleotide binding. The activity was assayed at low concentrations of ADP-Glc (0.2 mM) in presence of 1 mM of Fru-1,6-bisP and different concentrations of NTP-Mg^2+^ complexes (Table 4). In good agreement with the results described above, the activity of the enzyme was inhibited by the increased concentration of NTP-Mg^2+^ (Table 4). Values of *I*_0.5_ (NTP-Mg^2+^ concentration giving 50% of the maximal inhibition), in decreasing order GTP > CTP > UTP > ITP > ATP, were of the same magnitude for the three four nucleotides (22-11 mM). For ATP this value was approximately 4-fold lower (Table 4). This indicated that there is no dramatic difference in apparent binding affinity for the nucleotides. Consequently, the big differences observed in catalytic efficiencies for ATP are due in great part to *k*_cat_ (Tables 1, 2).

**Table 4:** Inhibition of the pyrophosphorolysis activity with the different nucleotides.

	***I*_0.5_ (mM)**
ATP-Mg	3.1 ± 0.3
ITP-Mg	11.1 ± 0.3
UTP-Mg	12.7 ± 0.6
CTP-Mg	16.4 ± 0.5
GTP-Mg	22.2 ± 0.7

It may be expected that differences in the nucleotide base could lead to lower apparent affinities for the non-preferred substrates. However, all the nucleotides have a similar *S*_0.5_ in the presence of the activator (Table 1). In the absence of the activator, the preferred substrate ATP has a much higher *S*_0.5_ than the alternative ones (Table 1). A possible explanation is based on the known fact that non-productive binding could lower *K*_m_ and *k*_cat_ simultaneously (Fersht, 1999). If the population of non-productive conformations increases, the apparent affinity will increase but the overall rate will suffer. Hence, the hypothesis is that ATP binds primarily in a productive conformation, whereas the binding of non-preferred NTPs lead to a larger population of non-productive conformations. To explore the structural feasibility of this hypothesis, we conducted molecular dynamics simulations to evaluate whether these alternative nucleotides could bind in different conformations from ATP. It is important to note that ATP binds first in an ordered mechanism (Haugen and Preiss, 1979; Kleczkowski et al., 1993; Jin et al., 2005), so the second substrate is not needed for ATP to be placed in the active site. To analyze the mobility when the active site was fully occupied, we later simulated the presence of NDP-Glc (substrate in the reverse direction).

**Figure 4:**
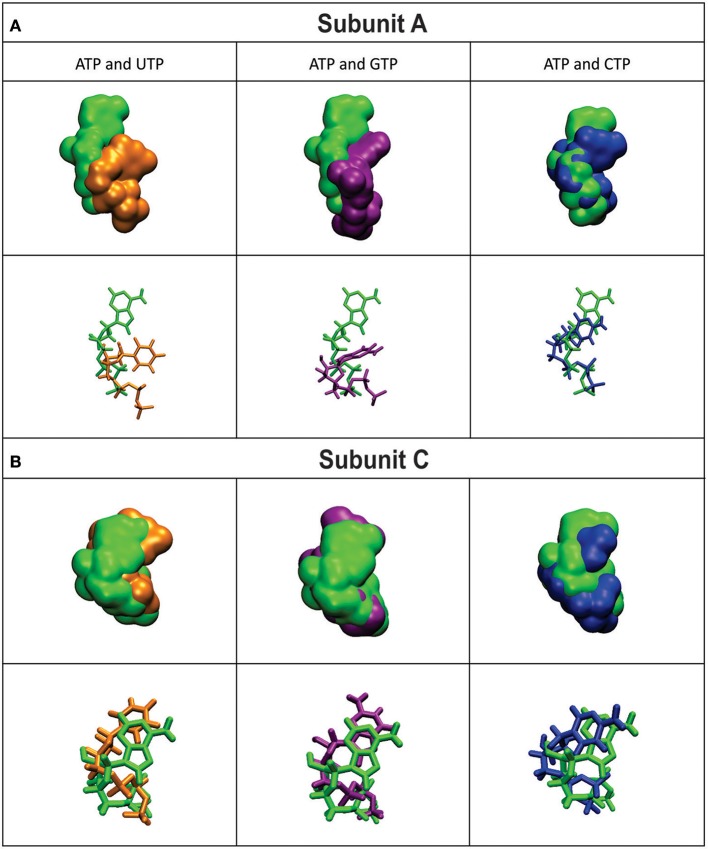
Density volumetric map of NTP Subunit A **(A)** and C **(B)**. Top-Volumetric map of ATP (green) over the last 5 ns of MD overlapped with the density volumetric map of NTP. Below-Average NTP structures calculated of the last 5 ns of MD overlapped with the average structures of NTP. Subunits B and D are not shown because they do not contain ligands. The template used to produce the models contains ATP in only subunits A and C.

**Figure 5:**
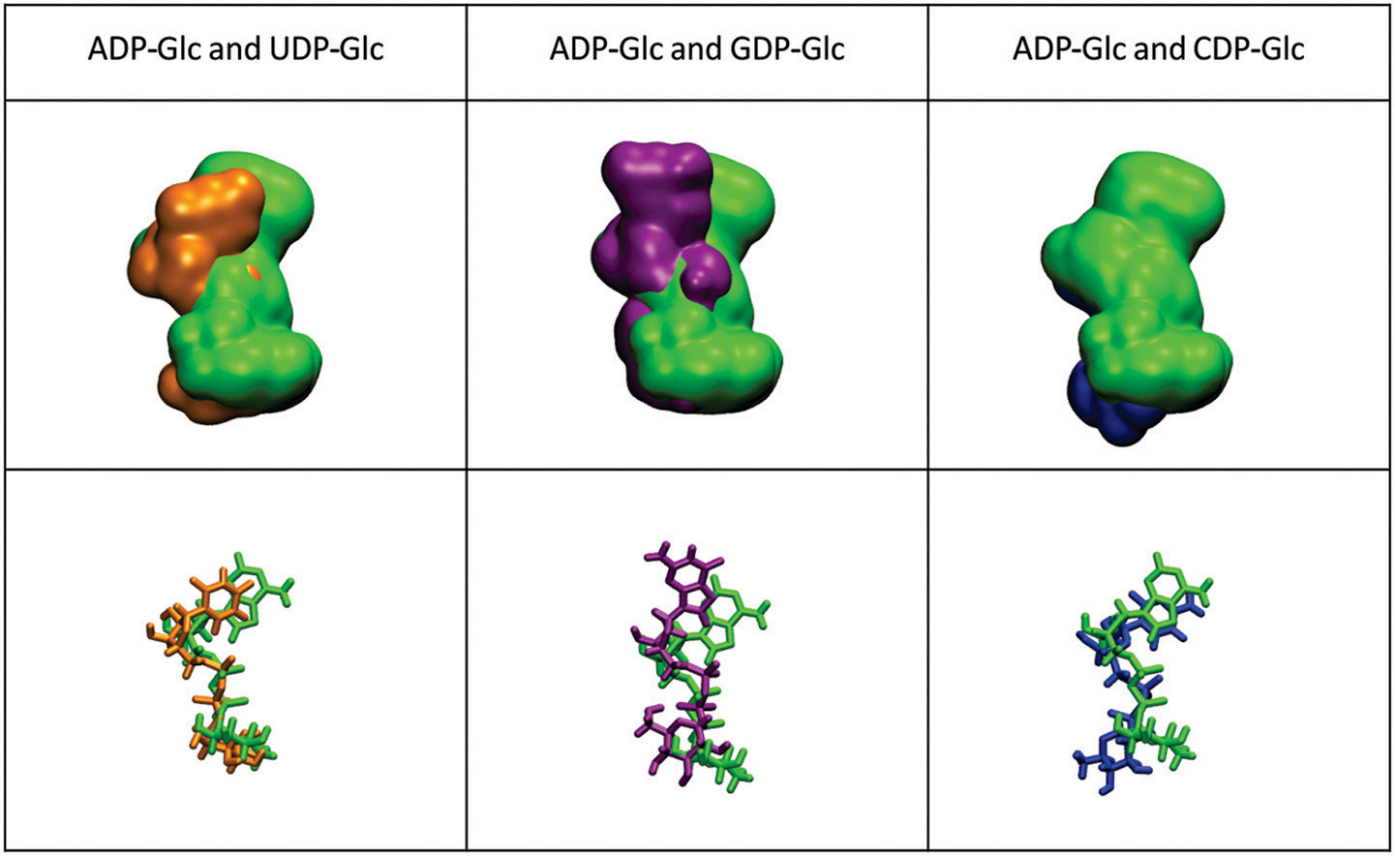
Density volumetric map of NDP-Glc. Top-Volumetric map of ADP-Glc (green) over the last 5 ns of MD overlapped with the density volumetric map of NDP-Glc. Below-average NDP-Glc structures calculated of the last 5 ns of MD overlapped with the average structures of NDP-Glc. The template used to produce the models contains ATP only in subunit A, which is the only one shown here.

The mobility of the four NTPs was visualized using volumetric maps of the atomic density. Figure 4 compares the map of weighted atomic density of each NTP. Beneath each volumetric map is the average structural representation calculated from the final 5 ns of the MD simulation. We observed in these simulations that alternative nucleotides bind with a much higher flexibility than the preferred substrate (ATP). Since there are a larger variety of conformations allowed for the alternative nucleotides, it is logical to assume that some will be non-productive. This would explain the *k*_cat_ and apparent *S*_0.5_ values for ATP: *k*_cat_ is higher because of a higher productive binding, and *S*_0.5_ is higher because a less flexible binding decreases the entropy, and as a consequence, the binding will seem to be less favored. The allosteric activation may improve the interaction between the protein and the substrate to lower the *S*_0.5_, which is observed for ATP when Fru-1,6-bisP is present (Table 1). A similar behavior is observed when the substrates of the reversed direction are analyzed (Table 2). In this case, there were also significant conformational differences during the simulation between the preferred substrate ADP-Glc and the other NDP-Glc variants (Figure 5).

## Discussion

Although enzyme specificity is fundamental in cell physiology and metabolism, most enzymes can promiscuously use different substrates from the ones they evolved to target. One way to prevent many of the undesirable outcomes of enzyme promiscuity is at the level of gene expression (Tremblay et al., 2006). In the present work, we show another type of control that occurs at the protein level via allosteric effects. For the *E. coli* ADP-Glc PPase this mechanism seems to be a simple way to control metabolic sorting. It is possible that the enzyme evolved its allosteric behavior not only to regulate glycogen synthesis, but also to acquire an effective manner to constrain its promiscuity.

ADP-Glc PPases have a common catalytic domain with other sugar nucleotide pyrophosphorylases (Jin et al., 2005; Cupp-Vickery et al., 2008; Cifuente et al., 2016). The formers generally have an extended C-terminal domain (120 to 150 amino acids) and a slightly longer N-terminal domain (10–40 amino acids). It is very possible that the fragment of ~150 amino acids at the C-terminus was acquired to create new regulatory roles and/or to improve a rudimentary regulation that was already present (Asencion Diez et al., 2013b). In addition, this newly acquired domain and its regulation could have worked as a tool to enhance substrate specificity. In other words, it could have favored metabolic regulation together with the choice of the correct substrate.

**Figure 6:**
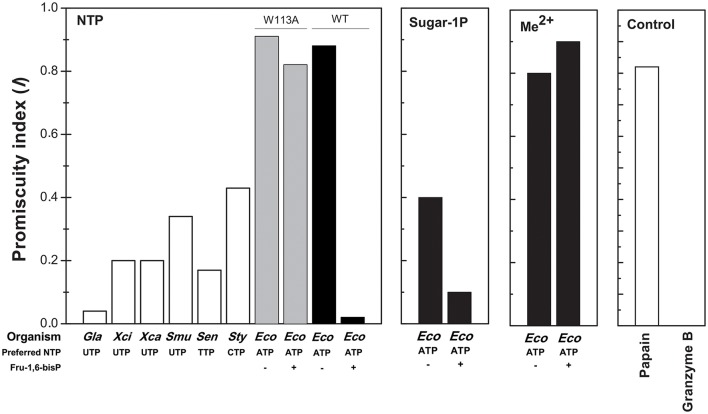
Promiscuity indices (*I*) of different enzymes. *I* for the use of NTPs was calculated, as indicated in Materials and Methods, for TDP-Glc PPase from *Salmonella enterica* (*Sen*); UDP-Glc PPases from *Xhantomonas campestri* (*Xan*), *X. citri* (*Xci*), *Streptococcus mutans* (*Smu*) and *Giardia lamblia* (*Gla*); CDP-Glc PPase from *S. typhi* (*Sty*); ADP-Glc PPase from *E. coli* (*Eco*, black bars) and the mutant *E. coli* ADP-Glc PPase W113A (*Eco*, gray bars). For the use of different sugar-1P and divalent cations, *I* values were calculated for *E. coli* ADP-Glc PPase (black bars). *I* values of highly promiscuous papain and almost completely specific granzyme B were included as control.

In the present work we found that Fru-1,6-bisP and Pyr improve the *k*_cat_/*S*_0.5_ of *E. coli* ADP-Glc PPase for ATP and also for Glc-1P as long as ATP is the co-substrate. This is a novel view of allosteric effectors as playing a key role in substrate selection. In good agreement with this model, the ADP-Glc PPase mutant W113A, which is insensitive to activation (Figueroa et al., 2011), has a similar *k*_cat_/*S*_0.5_ for all nucleotides tested in this work even in the presence of the allosteric activator. These results support the idea that a major effect of the allosteric regulator is to select the correct nucleotide (ATP) to favor the production of a specific sugar nucleotide (ADP-Glc) necessary for this particular metabolic pathway (glycogen synthesis). To quantify these effects, an index of promiscuity (*I*) has been proposed, which computes the degree of variability between different substrates (Nath and Atkins, 2008). As shown in Figure 6, in the presence of the activator, the ADP-Glc PPase turns from being a highly promiscuous enzyme to be a highly specific one.

Differences between promiscuous and native (preferred) activities can be manifested in differences in either *k*_cat_ or *K*_m_ (or *S*_0.5_). Specificity may result not only from substrate binding interactions *per se*, but also from appropriate positioning. Many promiscuous substrates have low *k*_cat_ values due to poor positioning relative to the active site's catalytic residues (Khersonsky and Tawfik, 2010; Nam et al., 2012; Donertas et al., 2016). This seems to be the case for ADP-Glc PPase, where the maximum turnover rate for ATP is greater but the apparent affinity is lower when compared to alternative NTPs. Binding of the native substrate is typically mediated by enthalpy-driven interactions, such as hydrogen bonds, whereas for the promiscuous substrates, hydrophobic and other entropy-driven interactions may play a role (Khersonsky and Tawfik, 2010). For instance, the existence of different alternative conformations may improve the apparent affinity. However, if alternative conformations are unproductive, substrates will exhibit low *k*_cat_ values. In our case, ATP is better positioned for catalysis than other nucleotides according to our MD simulations. This may imply a lower entropy and, as a consequence, a lower apparent affinity.

**Figure 7:**
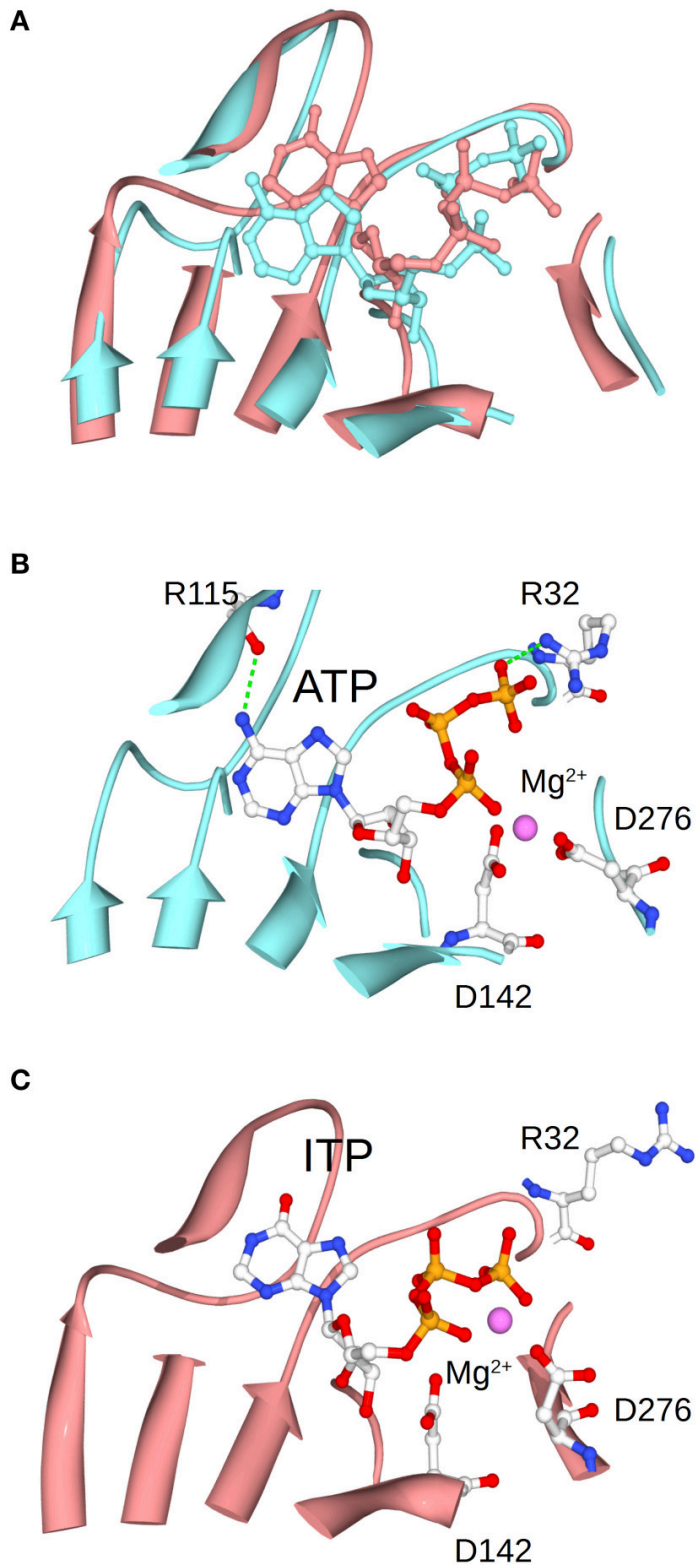
Positioning of ATP and ITP in the MD simulations. **(A)** Shows the overlap of the conformations of ATP and ITP in subunit A at the midpoint of the MD simulation. **(B,C)** Separately show the ATP and ITP, respectively. Structures were aligned based on the residues shown in the picture as ribbons, which are the one surrounding the substrate.

This improvement of specificity for ATP (mediated by Fru-1,6-bisP) seems to mainly occur through a dramatic change in the apparent affinity. In general, to enhance the specificity of the enzyme for a substrate, it is necessary to increase the number of specific interactions in the transition state (Fersht, 1999). In this particular case, these interactions, generated by the presence of the activator, should take place between the nucleotide base and the enzyme. This is because the only difference between the alternative substrates is found in the nucleotide base. For instance, ITP and ATP are identical, with the exception of a keto group vs. an amino group, respectively. This suggests that this amino group of the adenine base may contribute to the Fru-1,6-bisP driven increase in specificity and activation. MD simulations of the enzyme in presence of ITP show that this nucleotide varies its position when compared to ATP (Figure 7). The amino group of ATP makes a hydrogen bond with the backbone oxygen of Arg115, which is not possible for ITP. This hydrogen bond was experimentally observed in the ATP-bound x-ray structure of the potato tuber ADP-Glc PPase (Jin et al., 2005).

The frame of the MD simulation shown in Figure 7 illustrates how a slight misplacement of the substrate could affect catalysis. In panel C we observe that Arg32, which is critical for catalysis in the *A. tumefaciens* enzyme (Arg25) (Gomez-Casati et al., 2001), is not facing the substrate. In addition, Mg^2+^ is attracted by the γ- and β-phosphates of ITP, whereas the bivalent metal is chelated by critical residues Asp142 and Asp276 when ATP is present (Frueauf et al., 2001; Bejar et al., 2006). These differences between ATP and ITP were maintained throughout the simulation: the distance between the N of the amino group of ATP and the O of the peptide bond of Arg115 is mostly below 3 Å, whereas the analogous distance from the O of the keto group of ITP to the backbone of Arg115 is larger and more variable (~3.2–4.5 Å) (Figure S3). Crystal structures of the enzyme in the presence of different nucleotides will help to explain the binding differences.

All the previous results suggest that the effector would not only act as an activator, but also as a “selector” to improve nucleotide specificity. In addition, Fru-1,6-bisP affected the specificity for sugar-1P. The presence of this activator increased the *k*_cat_/*S*_0.5_ for all the monosaccharide-1P tested. Nevertheless, in the presence of Fru-1,6-bisP, the enzyme promiscuity index (*I)* was 4-fold lower (Figure 6). It has been described that the ADP-Glc PPase uses different metals as cofactor of the reaction (Preiss et al., 1966; Machtey et al., 2012; Asencion Diez et al., 2013a). Here, when the *E. coli* enzyme was assayed with UTP, it exhibited a greater activity in the presence of Co^2+^ and Mn^2+^ than with Mg^2+^ (Figure 2). Fru-1,6-bisP only affected the kinetic parameters when the reaction was performed with ATP, but there was no effect in increasing the specificity for the cofactor (Figure 6).

For ADP-Glc PPases a mechanism of allosteric activation has been described, in which hydrogen bond interactions between the loops associated with ATP binding would play a critical role (Figueroa et al., 2011; Hill et al., 2015). The Fru-1,6-bisP binds to the *E. coli* enzyme and transmits an allosteric signal to increase both the *k*_cat_ and the apparent affinity for the substrate ATP. Consequently, since this effect is only observed with ATP, Fru-1,6-bisP increases the specificity for this nucleotide favoring the synthesis of ADP-Glc. The fact that the amino group from ATP is important for activation and selectivity correlates with the role described for the loop Pro103-Arg115 (Figueroa et al., 2011, 2013; Hill et al., 2015). This amino group is surrounded by the loop (Jin et al., 2005; Figueroa et al., 2013), which implies the possibility that local conformational changes may improve its interaction with the enzyme. Further research is needed to confirm and understand the underlying mechanism.

The regulatory loop Pro103-Arg115 is a distinct insertional element only present in allosterically regulated sugar nucleotide pyrophosphorylases (Hill et al., 2015). This insertion could have been acquired as a tool for evolving both a regulatory role and a less promiscuous enzyme. The concept for controlling specificity by allosteric interactions may also apply to other enzymes. For instance, it has been described that in ribonucleotide reductase the specificity for NDP substrates is controlled by allosteric means (Ahluwalia et al., 2012; Hofer et al., 2012). Exploring this behavior in other enzymes will help to understand how broadly this phenomenon is in nature.

## Author contributions

Designed the research: MB Performed the experiments: AE, BH Performed computational research: LS, KO Analyzed the data: AE, AI, and MB. Contributed new reagents or analytic tools: AI, MB Wrote the paper: AE, AI, and MB.

### Conflict of interest statement

The authors declare that the research was conducted in the absence of any commercial or financial relationships that could be construed as a potential conflict of interest.

## Abbreviations

Glc-1P: glucose 1-phosphate
Gal-1P: galactose 1-phosphate
GlcN-1P: glucosamine 1-phosphate
ADP-Glc: ADP-glucose
UDP-Glc: UDP-glucose
GDP-Glc: GDP-glucose
Fru-1, 6-bisP, fructose-1: 6-bisphosphate.
