# Supplementary material for: Allosteric Control of Substrate Specificity of the Escherichia coli ADP-Glucose Pyrophosphorylase
Source: Front Chem. 2017 Jun 19;5:41. doi: 10.3389/fchem.2017.00041 (PMC5474683; doi:10.3389/fchem.2017.00041)
Supplement: Supplementary file 1 [file Table1.DOCX]

**Table S1. Promiscuity indices (*I*) for NTP use of different enzymes.**

| **Enzyme** | **NTP** | ***k*_cat_/*S*_0.5_ (s^-1^mM^-1^)** | ***I*** |
| --- | --- | --- | --- |
| *Sen*TDP-GlcPPase | TTP | 103 | 0.17 |
|  | UTP | 9 |  |
|  | GTP | 0.02 |  |
|  | CTP | 0.01 |  |
|  | ATP | 0.01 |  |
| *Xci*UDP-GlcPPase | UTP | 267 | 0.20 |
|  | TTP | 28 |  |
|  | ATP | 0 |  |
|  | GTP | 0 |  |
|  | CTP | 0 |  |
| *Xca*UDP-GlcPPase | UTP | 190 | 0.20 |
|  | TTP | 19 |  |
|  | ATP | 0 |  |
|  | GTP | 0 |  |
|  | CTP | 0 |  |
| *Smu*UDP-GlcPPPase | UTP | 51 | 0.34 |
|  | TTP | 16 |  |
|  | ATP | 0 |  |
|  | GTP | 0 |  |
|  | CTP | 0 |  |
| *Gla*UDP-GlcPPase | UTP | 329 | 0.04 |
|  | TTP | 11 |  |
|  | ATP | 0 |  |
|  | GTP | 0 |  |
|  | CTP | 0 |  |
| *Sty*CDP-GlcPPase | CTP | 36 | 0.43 |
|  | UTP | 50 |  |
|  | TTP | 0 |  |
|  | GTP | 0 |  |
|  | ATP | 0 |  |
| *Eco*ADP-GlcPPase W113A | ATP | 0.7 | 0.91 |
|  | UTP | 0.3 |  |
|  | TTP | 0.1 |  |
|  | GTP | 0.2 |  |
|  | CTP | 0.3 |  |
| *Eco*ADP-GlcPPase W113A  + Fru-1,6-bisP | ATP | 1 | 0.82 |
|  | UTP | 0.3 |  |
|  | TTP | 0.1 |  |
|  | GTP | 0.2 |  |
|  | CTP | 0.3 |  |
| *Eco*ADP-GlcPPase | ATP | 1 | 0.88 |
|  | UTP | 0.3 |  |
|  | TTP | 0.1 |  |
|  | GTP | 0.2 |  |
|  | CTP | 0.2 |  |
| *Eco*ADP-GlcPPase  + Fru-1,6-bisP | ATP | 177 | 0.02 |
|  | UTP | 0.3 |  |
|  | TTP | 0.1 |  |
|  | GTP | 0.2 |  |
|  | CTP | 0.2 |  |
