# Supplementary material for: Allosteric Control of Substrate Specificity of the Escherichia coli ADP-Glucose Pyrophosphorylase
Source: Front Chem. 2017 Jun 19;5:41. doi: 10.3389/fchem.2017.00041 (PMC5474683; doi:10.3389/fchem.2017.00041)
Supplement: Supplementary file 2 [file Table2.DOCX]

**Table S2. Promiscuity indices (*I*) for sugar-1P use of *E. coli* ADP-GlcPPase**

| **Enzyme** | **Sugar-1P** | ***k*_cat_/*S*_0.5_ (mM^-1^min^-1^)** | ***I*** |
| --- | --- | --- | --- |
| *Eco*ADP-GlcPPase | Glc-1P | 18 | 0.40 |
|  | Gal-1P | 0.4 |  |
|  | GlcN-1P | 0.1 |  |
| *Eco*ADP-GlcPPase  + Fru-1,6-bisP | Glc-1P | 1888 | 0.10 |
|  | Gal-1P | 6 |  |
|  | GlcN-1P | 33 |  |
