# Supplementary material for: Allosteric Control of Substrate Specificity of the Escherichia coli ADP-Glucose Pyrophosphorylase
Source: Front Chem. 2017 Jun 19;5:41. doi: 10.3389/fchem.2017.00041 (PMC5474683; doi:10.3389/fchem.2017.00041)
Supplement: Supplementary file 3 [file Table3.DOCX]

**Table S3. Promiscuity indices (*I*) for cofactor use of *E. coli* ADP-Glc PPase**

| **Enzyme** | **Cofactor** | ***k*_cat_/*S*_0.5_ (mM^-1^min^-1^)** | ***I*** |
| --- | --- | --- | --- |
| *Eco*ADP-GlcPPase | Mg^2+^ | 0.4 | 0.80 |
|  | Mn^2+^ | 0.6 |  |
|  | Co^2+^ | 2 |  |
| *Eco*ADP-GlcPPase  + Fru-1,6-bisP | Mg^2+^ | 29 | 0.90 |
|  | Mn^2+^ | 37 |  |
|  | Co^2+^ | 72 |  |
