# Supplementary material for: Allosteric Control of Substrate Specificity of the Escherichia coli ADP-Glucose Pyrophosphorylase
Source: Front Chem. 2017 Jun 19;5:41. doi: 10.3389/fchem.2017.00041 (PMC5474683; doi:10.3389/fchem.2017.00041)

Figure S1. MD simulation stabilization. Simulations were performed as described in Materials and Methods with the ligands ATP or ITP, respectively.

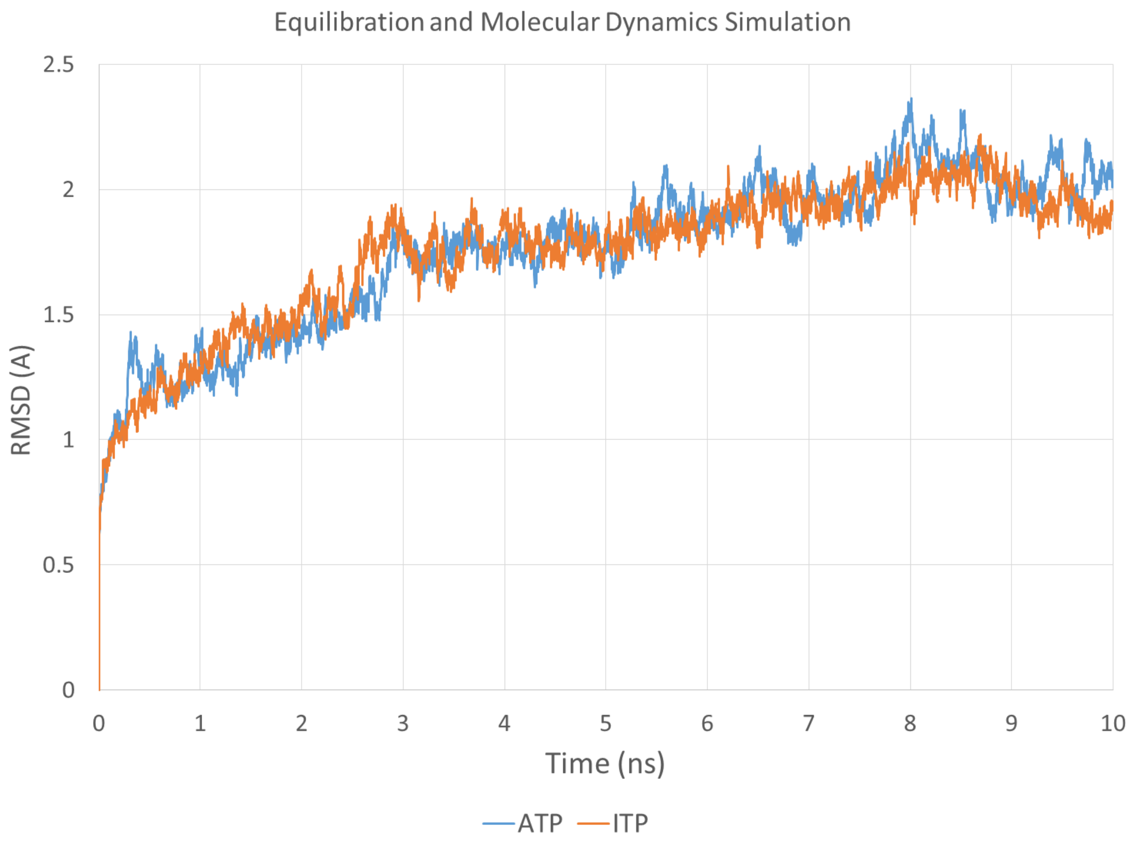

Supplement: Supplementary file 4 [file Image1.PDF]
