# Supplementary material for: Allosteric Control of Substrate Specificity of the Escherichia coli ADP-Glucose Pyrophosphorylase
Source: Front Chem. 2017 Jun 19;5:41. doi: 10.3389/fchem.2017.00041 (PMC5474683; doi:10.3389/fchem.2017.00041)

Figure S2. Catalytic efficiencies for alternative substrates

E. coli ADP-Glc PPase activity was measured in presence of 1 mM Fru-1,6-bisP (white bars) or no effector (oblique line bars) with alternative (A) NTPs, (B) sugar-1-phosphates or (C) bivalent cations.

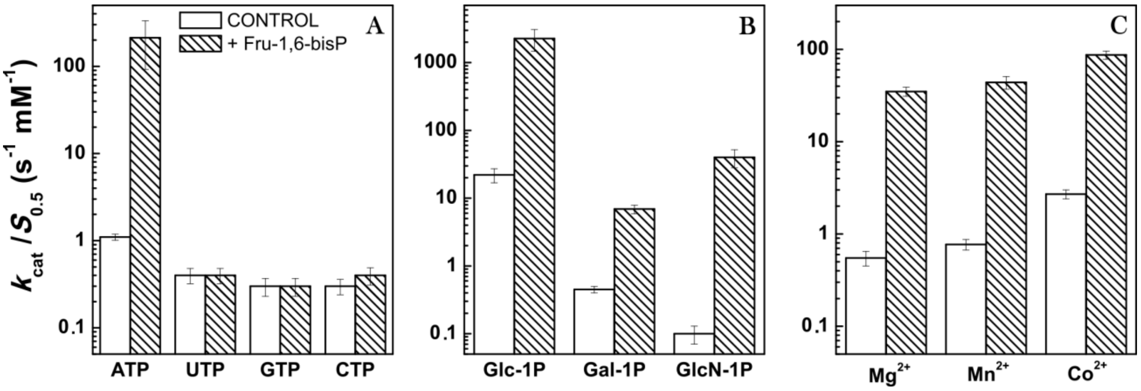

Supplement: Supplementary file 5 [file Image2.PDF]
