# Supplementary material for: Allosteric Control of Substrate Specificity of the Escherichia coli ADP-Glucose Pyrophosphorylase
Source: Front Chem. 2017 Jun 19;5:41. doi: 10.3389/fchem.2017.00041 (PMC5474683; doi:10.3389/fchem.2017.00041)

Figure S3.Location of ATP and ITP in the ADP-Glc PPase from *E. coli*. Simulations were performed as described in Materials and Methods with the ligands ATP or ITP, respectively. Blue represents the distance between the N of the amino group of ATP and the O of the peptide bond of Arg115. Orange represents the distance between the O of the keto group of ITP and the O of the peptide bond of Arg115. Only the last 5 ns of the simulation are plot. In the second panel, the distribution of distances is plot.

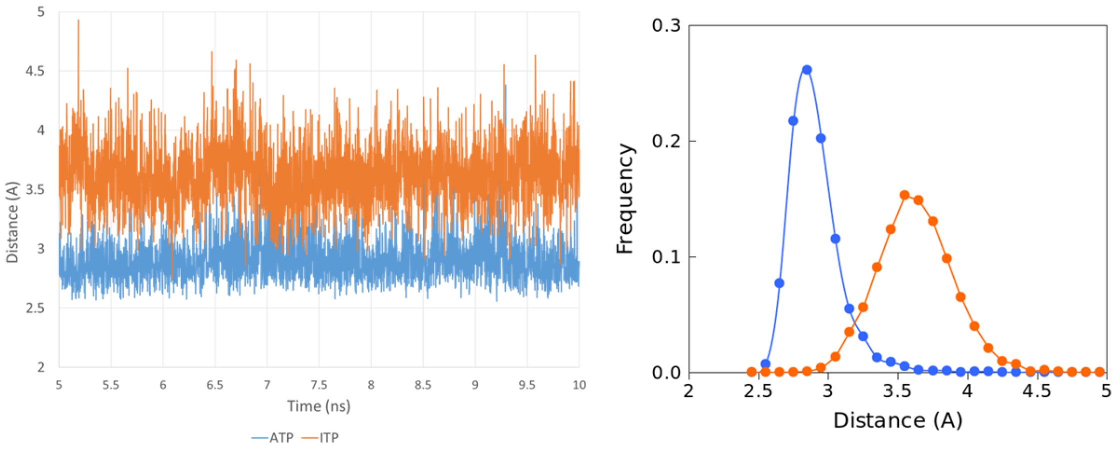

Supplement: Supplementary file 6 [file Image3.PDF]
